# NTM-Forum „Von der Abschaffung der Wissenschaften. Zur Geschichte und Zukunft des Mittelbaus in der Wissenschafts‑, Medizin- und Technikgeschichte“

**DOI:** 10.1007/s00048-024-00390-5

**Published:** 2024-07-12

**Authors:** Christian Sammer, Janina Wellmann, Carola Oßmer, Christian Zumbrägel

**Affiliations:** 1 Institut für Geschichte und Ethik der Medizin, Ruprecht-Karls-Universität Heidelberg, Im Neuenheimer Feld 327, 69120 Heidelberg, Deutschland; 2 Max-Planck-Institut für Wissenschaftsgeschichte, Boltzmannstr. 22, 14195 Berlin, Deutschland; 3 Institut für Philosophie, Literatur‑, Wissenschafts- und Technikgeschichte, Technische Universität Berlin, Straße des 17. Juni 135, 10623 Berlin, Deutschland

In den letzten Jahrzehnten ist die deutsche Universität auf die Glaubenssätze des New Public Management hin ausgerichtet worden, zu dessen obersten Zielen Innovation gehört. Innovation, so die Rhetorik, sei das Ergebnis von Flexibilität. Diese diene nicht allein dem Erhalt der Wettbewerbsfähigkeit der deutschen Wissenschaft, vielmehr ermögliche die herrschende Projektlogik eine agile Forschung, adaptiv und nah an den sich beständig wandelnden Bedürfnissen einer dynamischen Gesellschaft. Deutsche Wissenschaftler:innen befinden sich – zweifellos ein Alleinstellungsmerkmal – in der systematisch auf Unsicherheit und Unplanbarkeit hin optimierten flexiblen Förderung. Seit Jahren prägt die Praxis der befristeten Beschäftigung das Leben von Promovierenden, Postdocs, Habilitierten und bisweilen auch Professor:innen, die an den Universitäten und Forschungseinrichtungen des Landes in Dauerschleife Projekte bearbeiten, zugleich aber in Forschung, Lehre und Administration Daueraufgaben stemmen. Argumentativ gestützt wird diese Situation, indem die fortwährende Bewährung durch Wettbewerb als notwendig erklärt wird, bis durch Bestenauslese die Spitze – die Professur – erklommen oder verdientermaßen verfehlt worden sei. Vermeintlicher Erfolg wird den Mitgliedern des Wissenschaftsbetriebs in unterschiedlichsten Rankings auf der Grundlage von zweifelhaften Metriken zugeschrieben, jedoch mitnichten gemessen. Die Vielzahl unterschiedlicher Rankings trägt dementsprechend dazu bei, einen Ungleichheiten (re-)produzierenden Wettbewerb mit seinen Sieger:innen und Verlierer:innen als zentralen politischen Referenzrahmen zu institutionalisieren (Mau 2017: 71–92). Damit werden zum einen systeminhärente Risiken auf die Wissenschaftler:innen übertragen. Weil Erfolgsmetriken jedoch „irgendwann zum Selbstzweck werden“ (Baudson & Altieri 2023: 70), operationalisieren sie zum anderen Wissenschaft selbst als ein System, das als Quasi-Markt nach ökonomischen Kriterien funktionieren soll (Dartenne 2023).

Zu den Folgen einer solchen Begeisterung für Wissenschaft als Wettbewerb zählen ein ausuferndes, sich selbst reproduzierendes Antragswesen, das Forschungsergebnisse oft genug simuliert statt produziert, personelle wie materielle Kapazitäten der Hochschulverwaltungen bindet und einen rechtlich kaum noch zu überblickenden Dschungel schafft; eine Hochschullandschaft, in der sich zwischen Forschung und Lehre ein Hiatus aufmacht, der es verkompliziert, ein breites, stabiles und vor allem nachhaltiges Lehrangebot anzubieten; ein System, das Misstrauen sät, wenn nicht gar toxische Strukturen befördert und dazu führt, dass motivierte Forscher:innen ihrem Beruf den Rücken kehren, sodass lang aufgebaute hochspezialisierte Expertisen verloren gehen; eine Wissenschaft, die Kontinuität und Weitblick vermissen lässt und schließlich – wenngleich kapitalistisch organisiert – statt die avisierten Höchstleistungen zu produzieren, geistig verarmt (Bahr et al. 2022: 63–96).

Der politische Wille scheint groß, auf diese qualifizierten Wissenschaftler:innen verzichten zu wollen oder zu können. Während sich etwa Dieter Lenzen als Hanno unlängst in der *F.A.Z.* für die Mechanismen „psychosozialer Selektion“ (Lenzen 2023) vergangener Zeiten dankbar zeigte und die Sonne der Selektion erstrahlen ließ, kommt dem:der ein oder anderen in der Gegenwart Beheimateten wohl eher das Bild der Titanic in den Sinn, wo die Anzahl der Rettungsboote schlicht nicht für alle Passagiere ausreicht. Die Versuche der letzten siebzehn Jahre, dieses System zu reformieren, zementierten nur diese Schieflage zulasten der Mehrheit seiner relevanten Mitarbeiter:innen. Das 2007 in Kraft getretene Wissenschaftszeitvertragsgesetz (WissZeitVG) entkoppelte zunächst befristete Anstellungen von Sachgründen, stärkte nach seiner Novellierung 2016 dann die Begründung, zur Qualifikation speziell befristet beschäftigen zu dürfen, und schuf bei der Umsetzung mehr Verwirrung als Klarheit. Im Jahr 2021 hat sich die Ampelregierung in ihrem Koalitionsvertrag das Ziel gesetzt, das WissZeitVG zu reformieren. Im März 2023 veröffentlichte das von der FDP geführte Bundesministerium für Bildung und Forschung erstmals ein Eckpunktepapier. Das darin erklärte Problem, Postdoktorand:innen erführen in ihrer Erwerbsbiografie zu spät von der Chancenlosigkeit ihrer Festanstellung im Wissenschaftssystem, wurde mit dem Vorschlag pariert, die Höchstbefristungsdauer nach der Promotion von jetzt sechs auf drei Jahre abzusenken. Die Pläne riefen unter Wissenschaftler:innen aller Statusgruppen und Fachdisziplinen eine Welle des Protests hervor, woraufhin der Entwurf zurück in die „Montagehalle“ geschickt wurde. Der revidierte Referent:innenentwurf machte aus diesen drei Jahren eine Kombination von vier plus zwei Jahren: Nach vier Jahren soll eine weitere Befristung von maximal zwei Jahren nur noch mit einer Entfristungszusage möglich sein. Überzeugen konnte diese Reform der Reform die Stakeholder nicht, griff sie auch keinen der „konstruktiven Vorschläge“ aus dem akademischen Feld auf, die „auch auf explizite Aufforderung des BMBF“ vorgebracht worden waren (Bischof et al. 2024: 3). Einigung fand dieser Vorschlag nicht mal in der Ressortabstimmung – und dennoch schickte das Bundeskabinett Ende März 2024 diesen mit großer Skepsis begleiteten Entwurf ins parlamentarische Gesetzgebungsverfahren (BMBF 2024; Wiarda 2024).

Die Jahrestagung der Gesellschaft für Geschichte der Wissenschaften, der Medizin und der Technik (GWMT) widmet sich 2024 dem Thema „Wissenschaft und Aktivismus“. Wir verstehen das Forum zur Geschichte, Gegenwart und Zukunft des Mittelbaus an Hochschulen in Deutschland als einen polyphonen politischen wie auch wissenschaftlichen Beitrag dazu, denn auch in der Wissenschafts‑, Technik- und Medizingeschichte drängt das Problem der universitären Beschäftigung im Mittelbau auf eine Lösung. Wie in anderen „kleinen Fächern“ ist die Anzahl der Lehrstühle übersichtlich, zudem sind die wenigen Fachstandorte geografisch weit gestreut. Das bedeutet einen starken Wettbewerb, fordert von den Beschäftigten im Mittelbau ein hohes Maß an Flexibilität und produziert sogenannte „Schweinezyklen“: In kleinen Fächern kommt es gehäuft vor, dass entfristete Stellen, zumeist Professuren (deren Wiederbesetzung zudem fragiler ist als in den großen Fächern), auf Jahre hin schlicht nicht zur Verfügung stehen. Die Folge sind generationsspezifische Risiken. Dass ein Nachwuchsmangel sich in diesen Fächern daher besonders deutlich abzeichnet, kann kaum überraschen (Arbeitsstelle Kleine Fächer 2022). Die Institutsberichte der GWMT zeigen zudem eine düstere Befristungslage in der Wissenschafts‑, Technik- und Medizingeschichte. Die seit 2017 jährlich durchgeführte vertrauliche Befragung über die Beschäftigungsbedingungen in diesen Disziplinen macht deutlich: Im Durchschnitt der letzten Jahre schwankte die in den Institutsberichten angegebene Quote der im Mittelbau befristeten Medizin‑, Wissenschafts- und Technikhistoriker:innen zwischen 79 und 87 Prozent. In der Umfrage des Jahres 2023 lag der Anteil der angegebenen promovierten und nichtpromovierten befristet Beschäftigten auf Drittmittel- oder Haushaltsstellen bei 84 Prozent und somit mit Blick auf die zu stemmenden Daueraufgaben an Universitäten – allen voran die Lehre – „viel zu hoch“ (Bock von Wülfingen et al. 2023: 32).

Das „Forum“ in diesem Heft greift die Situation des Mittelbaus auf, um sie für unsere Fächer zu diskutieren und historisch einzuordnen, um die gegenwärtigen Verhältnisse kritisch zu verorten und zugleich langfristige Pfadabhängigkeiten prekärer Arbeitsbedingungen und Wissensökonomien aufzuzeigen. Das Forum versammelt unterschiedliche kurze Beiträge, die sich dem Thema aus wissenschaftlicher und persönlicher, politischer oder historischer Perspektive nähern. Die Beiträge präsentieren in ihrer Zusammenstellung Beispiele dafür, wie sich die Situation des Mittelbaus aus verschiedenen Blickrichtungen heraus historisch erforschen und darstellen lässt, die Fallbeispiele stehen aber keinesfalls repräsentativ für die Situation des Mittelbaus im Allgemeinen: Intersektionale Perspektiven werden in diesem Forum höchstens angedeutet. Wie die systematische Bevormundung und Prekarisierung des Wissenschaftssystems gegenüber seinen nicht professoralen Mitgliedern sich auf Nicht-Männer, Nicht-Deutsche, Nicht-Weiße oder Nicht-bürgerliche Personen auswirkte, bleibt hier unterbeleuchtet. Um diese Lücken zu schließen, bräuchte es weitere empirische Studien, die für verschiedene Wissenschaftskontexte komplexere und vergleichbare Entwicklungen aufzeigen. Wir hoffen jedoch, dass das Forum Impulse setzen kann, um diese wichtige Leerstelle zukünftig anzugehen.

Unter dem Titel „It’s the history, stupid“ zeigt der erste Beitrag von Matthias Berg die historischen Beschränkungen auf, aus denen sich die Diskussion und der (Un‑)Wille zu Reformen jenes Unwesens von Assistent:innentum, Privatdozentur oder Nicht-Ordinarien bis heute nicht zu befreien vermag. Dieses Übel hat bereits Max Weber zu Beginn des letzten Jahrhunderts als „Hasard“ charakterisiert, das sich wenig überraschend als nicht exportfähig erweisen sollte. Nicht nur die Privatdozentur, auch die Habilitation gehört zu jenen deutschen Eigenarten, die wissenschaftliche Aspirant:innen in die Universität einbindet. Einerseits alles andere als unproblematisch, thematisiert Reinhard Kahle die Habilitation andererseits in Anbetracht ihrer „Abschaffung“ beziehungsweise ihres Ersatzes durch Juniorprofessuren oder äquivalente Leistungen und findet zu einem bedenkenswerten „Lob der Habilitation“, gerade mit Blick auf die Zukunft kleiner Fächer wie der Wissenschafts‑, Medizin- und Technikgeschichte.

Die Habilitation nicht nur als mögliches Auslaufmodell, sondern als Krise der Universitätsentwicklung schon in den 1980er und 1990er Jahren untersucht Peer Pasternack in seinem Beitrag „Zwei Vorläufer der aktuellen Beschäftigungskrise“. Auch damals zeigten sich die „Aufstiegskanäle verstopft“, worauf die westdeutsche Gesellschaft indes mit einem beispiellosen Umbau des Wissenschaftssystems im wiedervereinten Deutschland reagierte. Einen persönlichen Einblick in die Kohorte, die in dieser Zeit nach der Bildungsexpansion in das Wissenschaftssystem einrückte, gewährt Ralf Forsbach, der diese Gruppe der „Boomer der Bonner Republik“ untersucht. Obwohl sie „Profiteur:innen der Sozial- und Bildungsreformen“ waren, setzte sich der Eindruck fest, immer zu viele zu sein, auch im Wissenschaftsbetrieb zu wenig Platz vorzufinden, sich von einer Finanzierung zur nächsten zu hangeln, während „Bundes- und Landesregierungen weder rechts noch links der Mitte die Probleme der wissenschaftlichen Mitarbeiterinnen und Mitarbeiter auch nur wahrnahmen.“

Dieses Urteil wohl teilend, werfen Amrei Bahr, Kristin Eichhorn und Sebastian Kubon einen prägnanten Blick auf die bisherigen Entwicklungen und möglichen Weiterentwicklungen ihrer im Jahr 2021 angeschobenen Initiative #ichbinhannah. Unter dem Titel „Wie wir dort gelandet sind, wo wir jetzt wegmüssen“ bilanzieren sie: Es brauche eine nachhaltige Transformation des aktuell dysfunktionalen Wissenschaftssystems, um sich gegen die von außen einwirkenden multiplen Krisen zu wappnen und die „disruptive Kraft“ der Wissenschaften wiederzugewinnen.

Wie wenig reformaffin sich das deutsche Hochschulsystem gleichwohl bereits in der Vergangenheit gezeigt hat, verdeutlicht die Analyse von Heiko Stoff, die sich spezifisch dem Umbau der Hochschulmedizin widmet. Bereits mit der Gründung medizinischer Akademien in den 1960er Jahren wurde eine Umstrukturierung versucht, die aber an „stabilen Subordinationsverhältnissen“ scheiterte. Wie sich der Mittelbau schließlich selbst gegen existierende Strukturen der Unterordnung wehrt und gewehrt hat, um sich aus der Situation des subalternen ewigen Nachwuchses oder „Taschenträgers“ zu befreien, schildern die Beiträge über die Erfolgsgeschichte der AG-Mittelbau – vom Mittelbau-AG-Kollektiv selbst verfasst – und die Etablierung des Driburger Kreises als lebendige „Institution ohne Institutionalisierung“ von Thorsten Bendl, Gina Maria Klein und Alexander Stöger.

Wo lässt uns dieses Forum nun zurück? Es zeigt in seiner knappen Vielstimmigkeit auf, zu welchem Preis das gegenwärtige Wissenschaftssystem operiert und wer diesen zu zahlen hat: die befristet beschäftigten Promovierenden, Postdocs und Habilitierten, aber auch die Hochschullehrenden unserer Fächer, die eine Reform der Befristungspraxis ebenso unterstützen. Wir stehen mithin an einem Punkt, an dem politisches Engagement – Aktivismus – in eigener Sache notwendig ist. Dabei soll das Forum einen Beitrag zur laufenden Debatte leisten, indem es die entscheidenden Narrative, die unsere aktuelle Misere legitimieren sollen, historisch aufdeckt – sprich dekonstruiert: Wissenschaft ist etwas anderes als ein Beruf, und der Mittelbau nur ein Durchlauferhitzer, in dem man entweder als Kalkablagerung zurückbleiben und zum Wohle des Systems rasch entfernt werden müsse oder derart beschleunigt werde, dass der Olymp der Professur erreicht werden kann. Dass dies nicht stimmen kann, wird schnell klar, denn wo bliebe dann die ganze durch den Wettbewerb produzierte Bildungs- und Forschungselite ohne die Basis des Bildungs- und Forschungsbetriebs?
